# Supporting Practices to Adopt Registry-Based Care (SPARC): protocol for a randomized controlled trial

**DOI:** 10.1186/s13012-015-0232-2

**Published:** 2015-04-09

**Authors:** Rebecca S Etz, Rosalind E Keith, Anna M Maternick, Karen L Stein, Roy T Sabo, Melissa S Hayes, Purvi Sevak, John Holland, Jesse C Crosson

**Affiliations:** Department of Family Medicine and Population Health, Virginia Commonwealth University, 830 East Main Street, Room 629, PO Box 980101, Richmond, VA 23298-0101 USA; Mathematica Policy Research, Princeton, NJ USA

**Keywords:** Population health, Diabetes, Disease registry, Implementation, Primary care, Sustainability

## Abstract

**Background:**

Diabetes is predicted to increase in incidence by 42% from 1995 to 2025. Although most adults with diabetes seek care from primary care practices, adherence to treatment guidelines in these settings is not optimal. Many practices lack the infrastructure to monitor patient adherence to recommended treatment and are slow to implement changes critical for effective management of patients with chronic conditions. Supporting Practices to Adopt Registry-Based Care (SPARC) will evaluate effectiveness and sustainability of a low-cost intervention designed to support work process change in primary care practices and enhance focus on population-based care through implementation of a diabetes registry.

**Methods:**

SPARC is a two-armed randomized controlled trial (RCT) of 30 primary care practices in the Virginia Ambulatory Care Outcomes Research Network (ACORN). Participating practices (including control groups) will be introduced to population health concepts and tools for work process redesign and registry adoption at a meeting of practice-level implementation champions. Practices randomized to the intervention will be assigned study peer mentors, receive a list of specific milestones, and have access to a physician informaticist. Peer mentors are clinicians who successfully implemented registries in their practices and will help champions in the intervention practices throughout the implementation process. During the first year, peer mentors will contact intervention practices monthly and visit them quarterly. Control group practices will not receive support or guidance for registry implementation. We will use a mixed-methods explanatory sequential design to guide collection of medical record, participant observation, and semistructured interview data in control and intervention practices at baseline, 12 months, and 24 months. We will use grounded theory and a template-guided approach using the Consolidated Framework for Implementation Research to analyze qualitative data on contextual factors related to registry adoption. We will assess intervention effectiveness by comparing changes in patient-level hemoglobin A1c scores from baseline to year 1 between intervention and control practices.

**Discussion:**

Findings will enhance our understanding of how to leverage existing practice resources to improve diabetes care in primary care practices by implementing and using a registry. SPARC has the potential to validate the effectiveness of low-cost implementation strategies that target practice change in primary care.

**Trial registration:**

NCT02318108

**Electronic supplementary material:**

The online version of this article (doi:10.1186/s13012-015-0232-2) contains supplementary material, which is available to authorized users.

## Background

Diabetes is the seventh leading cause of death in the United States and currently affects more than 25 million US adults, resulting in total related health costs of more than $170 billion annually [1-7]. Many diabetic complications could be prevented with improved management of glycemia, blood pressure, and lipids. However, this management remains suboptimal in many primary care settings where diabetes care suffers from ‘clinical inertia’ or the failure to appropriately intensify treatment by adding new treatments to achieve control of glycemia, hypertension, and dyslipidemia [8-17]. Because most patients with diabetes and other chronic conditions seek care from primary care practices, improving care in these settings will have a large impact on reducing diabetes-related excess morbidity and mortality and may also help control the costs of diabetes [18]. However, primary practices often lack an effective infrastructure for systematically monitoring their patients’ achievement of diabetes treatment goals to ensure that they are adhering to clinical guidelines [19].

The Chronic Care Model (CCM) is a population-based approach to care delivery that, after it is adopted, can enhance the abilities of primary care practices to improve adherence to clinical guidelines and outcomes by standardizing care delivery [20,21]. This process involves the implementation of registries, which allow practices to better track and monitor the care needs of their patients, providing the opportunity to identify and address clinical inertia among patients with diabetes [22]. The CCM supports the coordinated care that is a key aspect of the patient-centered medical home (PCMH) [23] model, the implementation of which has been shown to improve outcomes for patients with diabetes [24-26]. Unfortunately, a recent study of PCMH implementation in Virginia found that only 1% of practices were able to fully meet all PCMH requirements [27-29]. Most Virginia-based practices had introduced some elements of the PCMH model into their practice but had not focused on using the electronic health record (EHR) effectively for population-based care. For example, the study reported that only 33% of primary care practices in Virginia actively use their EHR for population management tasks such as those supported by registry usage [27-29]. This demonstrates that even those practices that have been able to achieve PCMH transformation have difficulty making population-based care a meaningful part of their everyday practice.

Transformational change that the implementation of CCM and PCMH requires often is overwhelming for primary care practices that are burdened by a payment system that has not consistently supported care management work. With recent changes to the US Medicare payment system (and to some systems of private payers as well), primary care practices will now have the opportunity to be compensated for chronic care management services [30,31]. Even with these changes to the payment system, however, many primary care practices do not have structures in place to easily make changes to care delivery or work processes to support monitoring of patients with chronic illness. For these PCMH and care management innovations to benefit patients with diabetes, these practices likely will struggle to adopt and implement new workflows that focus on addressing patient care needs and clinical inertia [24,27,32,33].

### SPARC: a low-cost intervention to facilitate population-based care

The Supporting Practices to Adopt Registry-Based Care (SPARC) intervention aims to help primary care practices in Virginia develop a population-based care delivery approach to improve care and outcomes for their patients with diabetes. SPARC provides basic support to practices to enable them to implement workflow changes to develop a diabetes registry and use it to manage and improve the care their patients receive. We define a diabetes registry as a searchable list of all patients in a practice who have type 2 diabetes that can be used to monitor patient records and identify gaps in care, lack of adherence to clinical guidelines, or potential instances of clinical inertia, to support patient outreach between scheduled office visits. Diabetes registries typically include clinical and administrative information. Clinical information commonly includes hemoglobin A1c levels, blood pressure, lipid levels, and preventive health screening information for all patients with diabetes. Administrative information varies more widely, but it often includes the date of each patient’s last visit and available demographic information, such as contact information, race and ethnicity, gender, and family supports. The SPARC intervention will focus on supporting practices’ development and use of diabetes registries for proactive care management to help patients achieve better diabetes control and prevent avoidable complications.

SPARC supports practices by advising them on registry development and helping them use existing resources to make workflow changes for successful registry implementation and use. Previous studies have focused on ensuring registry adoption through reliance on outside change agents, such as expert facilitators and evaluators [34-36]. However, interventions that rely exclusively on external resources require consistent funding support for these resources after the project ends. Helping practices use their own internal resources encourages sustainable practice-level innovation that can support problem-solving beyond the initial focus of this study on registry implementation and use. This approach also allows practice members to have complete control over their decisions, as well as flexibility to adapt the registry intervention so it will work best for their practice. Through the careful use of limited support resources, SPARC is designed to be low cost and to yield context-specific solutions, thereby increasing its replicability in a variety of settings. SPARC has the potential to affect the design of future interventions by validating the effectiveness of such low-resource implementation strategies that target practice change in primary care.

### Foundational research

Critical elements of the SPARC intervention were first identified through two previous studies. The Translating Research Into Action for Diabetes study found that improvements in processes of diabetes care (such as ensuring routine, periodic measurement of A1c) were not necessarily associated with improvements in intermediate clinical outcomes (such as A1c levels). Therefore, practice-level efforts to improve care for patients with diabetes will need to move beyond ensuring adequate monitoring alone to a more proactive focus on improving the management of care and appropriate treatment intensification for these patients—that is, a focus on acting on those measurements, when appropriate [37-39]. In addition, we pilot tested the feasibility of a low-cost approach to facilitate diabetes registry implementation and use through the Organizational Self-Assessment to Improve Diabetes Care in Primary Care Practices (R34DK075417) study, referred to hereafter as the R34 pilot study or the R34 study. In that study, six primary care practices worked to implement and use a diabetes registry. Four practices successfully implemented a diabetes registry and increased the percentage of their patients with HbA1c ≤ 7.0 from 77.4 to 79 (*p* = 0.001), with nonsignificant improvements in the percentage of patients with appropriate control of LDL and blood pressure.

Since the completion of the prior pilot study, there have been advances in EHR capabilities, and their use has continued to increase among primary care practices. For example, EHRs certified by the Office of the National Coordinator for Health Information Technology as meeting the standards for the Meaningful Use program sponsored by the Centers for Medicare and Medicaid Services must have registry functionality available as either a built-in function or an add-on module [40]. Therefore, practices participating in SPARC may be able to use EHR-based registry functions not available at the time of the previous pilot study. If participating SPARC practices have an EHR with registry functionality, the SPARC research team will encourage them to use that function. Otherwise, the research team will offer information on how to use a free manual registry software program.

### Study aims

The SPARC study will test a diabetes registry implementation intervention that provides low-intensity technical assistance and peer mentor support to primary care practice-level leaders as they focus on improving the quality of diabetes care in their practices. We will evaluate the effectiveness and cost-effectiveness of SPARC using the methods described below.

The SPARC study has the following aims:

- Aim 1: Conduct a randomized clinical trial to evaluate the effectiveness of a multifaceted work process redesign intervention for the implementation and use of a diabetes care registry in primary care practice.
- Aim 2: Evaluate the effect of the intervention on diabetes processes of care and patient outcomes.
- Aim 3: Evaluate the costs and cost-effectiveness of the intervention in primary care practices.
- Aim 4: Conduct a qualitative evaluation of the intervention implementation to fully understand the factors associated with success.

## Methods

SPARC is a two-armed RCT comparing the intervention condition of low-intensity technical assistance and peer mentor support for implementing a diabetes registry with the control condition of basic education on population health. We will evaluate the clinical effectiveness and cost-effectiveness of a low-cost intervention for implementing diabetes registries in primary care practices. This study was reviewed by the Virginia Commonwealth University (VCU) Institutional Review Board (IRB) and deemed exempt, because it presents minimal risk to participants and will not be collecting identifiable data on human subjects. SPARC is funded by the National Institute for Diabetes and Digestive and Kidney Diseases and is conducted jointly by researchers in the Department of Family Medicine and Population Health of VCU and Mathematica Policy Research. The New England IRB has reviewed and approved this study.

### Setting and recruitment

The intervention will target 30 Virginia internal medicine and family medicine practices participating in the Virginia Ambulatory Care Outcomes Research Network (ACORN) [41]. ACORN, sponsored by VCU, is a practice-based research network of nearly 100 primary care practices. Member practices are in rural, urban, and suburban settings and range from single-clinician practices to large practice groups and hospital-owned clinics. ACORN member practices choose which studies to be involved in and receive no specific benefit as part of their membership, other than the opportunity to contribute to advancing the science of primary care.

Practices eligible to participate in SPARC must meet the following criteria: (1) the practice must treat adult patients with type 2 diabetes, (2) the clinicians and staff in the practice must be able to change workflows in support of the intervention, and (3) the practice must have a clinician champion who can attend champion meetings (described below) and lead registry implementation in the practice. Practices already using a registry or those that do not currently use an EHR are excluded from participation.

### Randomization

To ensure a balance between the intervention and control groups of practices on the key practice characteristics of setting (rural or urban), size (fewer than three, versus three or more, clinicians), and ownership type (independent or group), the 30 practices were divided into the eight strata formed by these three characteristics and then randomized with equal probability between intervention and control using a random number generator, with 15 practices allocated to the intervention group and 15 to the control group. Each practice was assigned a study identification number upon allocation. Once a year, participating practices will provide the SPARC research team with the total number of adult patients with type 2 diabetes in their practice. Using that number as an upper threshold, a biostatistician on the SPARC research team will generate 100 random numbers for each practice. This number list will be used to identify 100 patient medical records for review in each practice.

### Sample size and power analysis

For evaluating intervention effectiveness, we will review 100 patient records in each of the 30 practices at baseline and 1- and 2-year follow-up intervals. We will draw independent random samples of patient records for each of these reviews, and data will be recorded without patient identifiers. Because patients are not recruited and are not the subjects of the intervention, patient dropout is not a significant issue.

The primary outcome variable is patients’ hemoglobin A1c levels. The research team obtained data on patient hemoglobin A1c levels from practices that participated in the R34 pilot, finding a mean of 7.1, a standard deviation of 1.6, and an intraclass correlation coefficient of 0.065, providing 80% power to detect a 0.5-level decrease in mean hemoglobin A1c from baseline to 1 year in the intervention arm as opposed to the control arm, allowing for up to 20% or six of the participating practices to drop out. If fewer practices drop out of the study, we will have 80% power to detect 15% improvement in the percentage of patients with LDL < 100 (from 53% at baseline to 61% at follow-up), a 20% increase in the percentage of patients with BP < 130/80 (42.2% baseline, 50.6% follow-up), and a 25% increase in the percentage of patients in each practice with HbA1c < 7 (59.2% to 74%). The anticipated baseline rates for these outcomes are derived from baseline rates observed in the pilot study practices.

### Participants

Each participating practice will identify two champions. One will be a clinician; the other could be anyone in the practice, including nonclinical staff, who could work regularly with a registry should one be implemented. Clinician champions must meet the following criteria: the clinician must be a medical doctor, doctor of osteopathy, physician assistant, or nurse practitioner; have their principal employment in the practice; be authorized to lead a practice improvement process; and regularly provide care to patients with type 2 diabetes.

### Intervention activities

Intervention and control practices will differ in the level of support and type of resources offered by the research team. All practices will participate in champion meetings at least twice during the project period. All practices will receive basic technical assistance if they are unable to identify their total patient population with type 2 diabetes. In addition to these activities, however, intervention practices will be paired with peer mentors and will be given access to continuing basic technical assistance offered by a physician informaticist with expertise in primary care data systems and reporting. Following the recommended criteria for specifying and reporting implementation strategies put forth by Proctor and colleagues, we specify the operationalization of our SPARC implementation strategy in Table 1 [42]. Next, we provide details of these project elements.

**Table 1:** **Specification of the SPARC intervention strategy**

**Specification domain**	**Specification of intervention strategy**
Actors	Peer mentors: clinicians who have implemented and maintained a diabetes registry in their practice
	Physician informaticists: clinicians with expertise in primary care data systems and reporting
Actions	1) Two champion meetings
	• Provide education to intervention and control practices on how to implement a diabetes registry, including:
	− Registry development
	− Population health in primary care delivery
	− The American Diabetes Association’s guidelines for patients with diabetes
	− Diabetes registry software options or communicating with EHR vendors about registry functionality
	− A practice self-assessment checklist designed to help practices plan and manage potential workflow changes
	• Facilitate discussion among intervention practices about the challenges faced during registry implementation and solutions developed to overcome those challenges
	• Provide intellectual space for intervention practices to process what they are learning and talk about their experiences with registry implementation, as well as develop a plan for sustaining or expanding their registry
	2) Peer mentoring
	• Advise intervention practices on registry implementation and using the materials disseminated at champion meetings.
	• Provide intervention practices with access to physician informaticists to assist with use of practice data systems.
Target of the actions	Practice champions: two champions from each intervention and control practice—one clinician champion and one champion who will be a potential user of a registry—will attend the champion meetings. Champions in intervention practices will work with the peer mentor.
Temporality and dose	1) Champion meetings
	• One champion meeting will be held before the intervention period and a second about 15 months later.
	2) Peer mentoring
	• Peer mentors will work with practice champions in intervention practices for the first 12 months of the intervention, maintaining monthly communication through telephone calls and practice visits.
Implementation outcome(s) effected	Change in mean patient hemoglobin A1c scores
Justification	We believe that helping practices use existing resources and learn how to solve problems to implement a diabetes registry and related workflow changes will be more sustainable than implementation strategies that rely more heavily on external resources.

### Champion meetings

Two champion meetings will be held for all participating practices. Control practices and intervention practices will meet separately. The first champion meeting will be largely the same for control and intervention practices. These meetings will provide an opportunity for participants to learn about diabetes registries, workflow redesign, and general principles regarding population-based approaches to care delivery for patients with chronic conditions. Participants will be introduced to a practice self-assessment checklist tool, designed during the R34 pilot project, to help practices plan workflow changes and registry goals. They will gain insights into what they need to discuss with their EHR vendor to make the registry work and will learn about software options available if their current EHR does not support a registry function. Champions will be introduced to the SPARC research team and will provide basic baseline demographic information on their practices and mix of patients. They will leave the meeting with a packet of information that includes the American Diabetes Association’s guidelines for patients with diabetes.

In addition to the champion meeting elements, intervention practices will receive two pieces of information not shared with control practices. First, they will be introduced to their peer mentors and will receive a document outlining basic implementation milestones (described below and in Additional file 1). Second, they will be introduced to a physician informaticist who will be available to them by telephone to answer basic questions during the study.

At the second champion meeting, 15 months later, participants will discuss the challenges they faced during registry implementation and solutions they developed to meet those challenges. This meeting will provide intellectual space for practices to process what they have learned and how they have changed during registry implementation. The research team will share preliminary findings from the first year with study participants. These will include patterns identified in the qualitative data by research analysts and changes in chart audit data from baseline to year 1. Practice members will be invited to help explain the significance of those findings. They will also be led in a discussion regarding plans to sustain, and for some expand, their now-existing registry.

### Peer mentors and physician informaticists

Peer mentors, available only to the intervention practices, are practicing clinicians who have successfully implemented and maintained a diabetes registry in their practice. The peer mentors will attend the first champion meeting for intervention practices to present information on registry development and answer questions about it. After the meeting, clinician champions from intervention practices will be paired with a peer mentor who will advise them on the use of the self-assessment tool and will offer basic guidance regarding workflow redesign. Peer mentors will visit each intervention practice at least twice during the first study year: once at baseline and once shortly after the practice begins using the registry. In between these visits, peer mentors will call practice-level champions in the intervention practices monthly to monitor implementation progress, offer assistance, and help the practice connect to basic technical assistance from the physician informaticist if needed. Practice-level champions can contact the peer mentors by telephone or email throughout the intervention period.

Some practices will be able to adopt registries successfully with little additional support; others may need some limited technical assistance or guidance. Peer mentors will tailor their efforts to specific practice situations to meet these varied needs. The mentors’ decisions regarding the need for additional in-person visits or more frequent telephone contact will be guided by the SPARC Milestones document, which outlines eight critical steps in the meaningful adoption of a diabetes registry (see Additional file 1). This document will also be shared with intervention practices during the first champion meeting. Peer mentors will use this document to assess monthly implementation progress for each intervention practice and determine whether a specific practice needs additional support.

Peer mentors will identify practices that would benefit from assistance from a physician informaticist as they implement their registry. The physician informaticists are primary care physicians with experience and expertise in primary care data systems and reporting who will be available to help practice champions with such tasks as querying existing records to populate stand-alone registry systems, identifying changes to work processes to ensure structured data entry of specific fields needed to populated integrated registry systems, and developing reports based on registry data.

An activity log of peer mentor and physician informaticist contacts and provision of basic technical assistance will be maintained to assess the level of effort provided to participating practices. These work logs will inform the cost and cost-effectiveness evaluation of the intervention, as described below.

### Data collection

SPARC researchers will collect data using mixed-methods explanatory sequential design [43]. In such a design, data collection happens in iterative cycles during which preliminary findings from one set of data influence data points collected in the next set of data. For example, findings during a chart audit review might identify the need for collection of qualitative data elements not previously identified, so that the significance of chart audit findings might be better understood. Data collected in SPARC will include medical record review, cost data, participant observations, key informant interviews, and in-depth interviews with practice champions and project leads [44]. Data collection will occur at baseline, 12 months post-registry implementation, and 24 months post-registry implementation. We understand it is possible that not all practices will successfully implement a diabetes registry and that some control practices may implement a registry without assistance. Based on our findings from the R34 pilot study, initial adoption of a registry will take up to 3 months. We will use medical record reviews (described in the next section) to collect patient outcome data (mainly hemoglobin A1c) with which we will evaluate the effectiveness of the registry. Data collected during qualitative site visits (described below) will allow SPARC researchers to evaluate the contextual factors and practice characteristics associated with successful implementation of a diabetes registry, including any barriers or problem-solving strategies that practices employ to enable registry implementation. During qualitative site visits, we will also collect information related to cost of the intervention in each practice.

### Medical record reviews

We will review the medical records of 100 randomly selected patients with diabetes from each participating practice to assess diabetes care quality at baseline, 12 months, and 24 months. These records will be selected from lists generated by practices at the start of the study period, as well as at each subsequent review point, of all patients with a visit coded with an ICD diagnosis code for type 2 diabetes, excluding any patients who are pregnant, under age 18, or over age 75. A trained medical record reviewer will visit practices to conduct reviews; reviews will be done with a structured abstraction or audit instrument. To provide quality assurance, a different staff member will review 10% of the already reviewed records. Medical record reviews will be standardized across all practices. In addition, each review may include up to five questions added at the participating practice’s request. During practice recruitment, practices were able to review the standardized chart review instrument and suggest additions most beneficial to their setting. If practices requested additional items, those items are added to the review of their practice information only. Data from medical record reviews will be directly entered into the study database using an electronic tablet-based instrument designed in SharePoint. In addition to data specified by the audit form, medical record reviewers will take notes regarding where in the record information was found and at what level of standardization within the practice.

### Qualitative site visits

SPARC researchers will visit each participating intervention and control practice to assess practice organizational characteristics and workflow related to care of patients with diabetes. Site visits will be conducted at three points during the study: at baseline, 12 months, and 24 months.

Researchers will use four primary methods for data collection during the site visits: participant observation, key informant interviews, cost survey instruments, and semistructured clinician interviews. Participant observations will consist of three to four observation periods in each practice, each period lasting 3 to 5 h. Observations will be guided by an observational template developed during the R34 pilot study and refined for use in this project. SPARC researchers will observe practice workflow, care delivery, and check-in processes, with special attention paid to diabetes-specific and registry-specific activities. During observations, researchers will identify two or three practice members for interviews to learn more about workflow changes in registry implementation. Qualitative site visits will also include interviews with practice managers or administrators to collect cost data relevant to registry implementation.

### Measuring registry cost

To estimate cost-effectiveness of the registry, we will compare costs experienced by intervention and control practices. At the 12- and 24-month site visits, SPARC researchers will collect practice-level ongoing operational costs in treating patients with diabetes. The instrument records the amount of time that clinicians, nurses, and office staff spend on patient encounters, chart reviews, and reports.

In addition to operational costs, the registry may entail start-up costs to practices. We will measure start-up costs, including purchases, staff training time, identification of patients for inclusion in a registry, and data entry during the baseline site visits. We will measure costs associated with use of peer mentors and physician informaticists by reviewing their activity logs.

We will estimate the dollar value of time spent by office staff and peer mentors using average wage rates by occupation, in Virginia, from the US Bureau of Labor Statistics.

### Outcomes

#### Statistical analysis

We will assess intervention effectiveness by comparing the change in patient-level hemoglobin A1c scores from baseline to year 1 between intervention and control practices. A linear mixed-effects model will be used to account for the continuous repeated-measure response (A1c), a fixed-effect group indicator (two levels: intervention, control), a fixed-effect time indicator (two levels: baseline, year 1), and an interaction of the group and time indicators. A practice-level random effect will be included to account for intracluster correlation. Any adjustments to this model for patient- or practice-level characteristics will be made by including those measures as fixed effects. We will analyze secondary effectiveness measures using similar linear mixed-effects models of numerical and categorical measurements, including whether diabetes patients met targets for blood pressure control or LDL cholesterol.

We will assess intervention maintenance of the change in A1c scores using a similar model, with the time periods changing to baseline and year 1, and year 1 to year 2. We will assess practice-level implementation and maintenance using *t*-tests for unadjusted comparisons and using analysis of covariance to adjust differences for practice characteristics. We will categorize practices according to their allocated group, regardless of compliance (intention-to-treat analysis) [45].

#### Cost analysis

We will calculate cost-effectiveness ratios by dividing the difference in intervention costs between the control and intervention groups by the difference in outcomes (for example, HbA1c) between the control and intervention groups. Results can be used to share cost-related information, such as the cost to a practice (or potential sponsor) for a .5 percentage point reduction in the mean A1c level across their patients with diabetes, with other practices. Because overall health care expenditures are lower for patients with good HbA1c control than for those with poor control, these practice-level intervention costs will also be compared to expected reductions in overall health care expenditures to estimate the potential societal impact of the intervention on health care expenditures [46].

#### Qualitative analysis

Analysis of qualitative site visit data will be conducted using grounded theory and a template-based analysis using the Consolidated Framework for Implementation Research [47]. SPARC researchers will identify practice characteristics (such as organizational structure), the practice’s ability to successfully implement a registry, and changes from registry implementation or consequences ascribed to it. SPARC researchers will create a codebook based on themes discovered in a preliminary reading of the data. The SPARC team will then code all available qualitative data from observations and interviews using ATLAS.ti. The team will use a consensus approach to create reliable and validated use of the codebook and will conduct quality assurance audits on 10% of all coded data. As a result of this process, some codes may be dropped and new codes added. After coding is complete, team members will begin the analysis process by highlighting major themes and relationships discovered throughout the coded data. The primary goals for this analysis are to (1) examine practices’ diverse responses to the intervention and (2) learn how practice characteristics shaped a practice’s response to the intervention. Qualitative data collected during the medical record review, concerning where data were found in the record and with what kind of practice-based standardization, will be included in this analysis.

### Trial status

Recruitment was conducted February to November 2014, with 30 practices currently enrolled in SPARC. The first champion meeting for control practices is scheduled to take place in March 2015, and we anticipate starting baseline data collection activities at that time. The first champion meeting for intervention practices will be held soon thereafter, with baseline data collection for that group beginning immediately after. Baseline data collection for control and intervention groups will be completed by June 2015. Data cleaning and analysis of preliminary baseline data will begin soon after baseline collection is complete.

## Discussion

As primary care practices around the country see a steady increase in the number of patients with chronic conditions, the importance of transforming work processes and adopting population-based approaches to care is heightened. However, many practices lack the internal flexibility or external support to integrate population-based care concepts into their everyday care processes. SPARC will test the effectiveness of a low-cost intervention designed to help primary care practices implement a population-based approach to care by developing a diabetes registry, while paying attention to work process change. All participating practices will have access to basic information on using population health approaches in primary care settings, developing registries, and preparing for work process change. Intervention practices will have additional assistance from peer mentors who have already developed and sustained a proactive diabetes registry in their practice.

The project period is 5 years, with 3 years for practice participation and data collection. The length of the project will enable the SPARC research team to determine the factors necessary for practices to successfully implement and sustain a diabetes registry. The SPARC research team also will be able to explore what other aspects of a practice are affected after registry implementation. For example, it is expected that practices that successfully implement a registry will see improvements in overall outcomes for their patients and increase standardization in medical record documentation.

SPARC is an innovative, low-cost intervention for transformational practice change. Previous studies, designed around high-cost interventions that rely on outside resources (such as expert facilitators), may be effective but lack replicability in everyday practice or sustainability. SPARC could show that using existing resources to support implementation can be effective and sustainable. In doing so, it could affect design of future interventions intended to guide primary care practice work process change.

## Additional file

Additional file 1:
**SPARC Milestones.** This document was shared with the intervention practices and outlines eight critical steps in the meaningful adoption of a diabetes registry.
