# Supplementary material for: Supporting Practices to Adopt Registry-Based Care (SPARC): protocol for a randomized controlled trial
Source: Implement Sci. 2015 Apr 9;10:46. doi: 10.1186/s13012-015-0232-2 (PMC4399225; doi:10.1186/s13012-015-0232-2)
Supplement: Additional file 1: — SPARC Milestones. This document was shared with the intervention practices and outlines eight critical steps in the meaningful adoption of a diabetes registry. [file 13012_2015_232_MOESM1_ESM.docx]

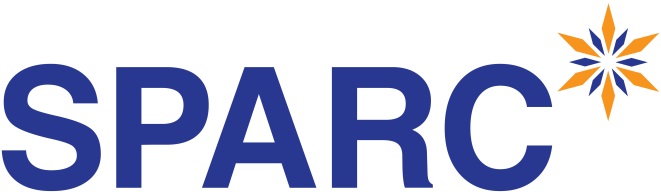
**Milestones**

A Stepped Process for Implementing a Diabetes Registry

# **Step 1: Identify SPARC Champions**

Who will lead the efforts to develop and implement a registry in your practice?

Identify two people in your practice who will serve as SPARC practice champions: one to act as lead and the other as a backup. The champions will lead the development of your registry, keep your practice on target for implementing the registry, plan logistics of going live with your registry, and help plan for your registry’s continued use. The champion also will be a contact person between your practice and the VCU research team during the study.

# **Step 2: Set Practice-Level Goals for Your Registry**

What goals would you like to achieve for your patients or your practice by implementing a diabetes registry?

1. **Brainstorm potential goals:** What patient outcomes would you like to affect? For example: (1) decrease the number of patients with type 2 diabetes who have an A1c of 7.0 or above, or (2) increase the number of patients with type 2 diabetes who receive nutritional counseling. When brainstorming, consider known drivers in your practice, such as clinicians commenting it is difficult to track X, patients frequently requesting Y information that is not easily found, or reporting needs for issues such as Meaningful Use.
2. **Choose goals** that matter the most to the clinicians in the practice.
3. **Choose goals** that matter the most to your patients.
4. **What are other clinicians’ goals?** Ask others in your practice about the goals they might have for patients who are on the registry.
5. **Prioritize practice goals** based on goals that you and others have selected.
6. **Set targets for attaining your practice goals** based on the information you gathered in steps 2.1–2.3. Share the targets you identify with other clinicians in your practice to make sure they appropriately match interest in your goals. This will be important for achieving buy-in from others in your practice. For example, a practice goal might be to decrease the number of patients with type 2 diabetes who have an A1c over 7.0. The target for this goal might be set at a 50 percent reduction within a year.
7. **Share the goals, targets, and timeline** with everyone in your practice.

# Step 3: Define Registry Content

How can you best use your registry to meet your goals?

**1. Identify measures to track** using current clinical guidelines and the targets that you identified in step 2.6. For example, you could start with the “ABCs of diabetes management from the National Diabetes Education Program” available at: <http://ndep.nih.gov/publications/PublicationDetail.aspx?PubId=114>

**2. Identify the patient population** to include in your registry at the start based on the practice goals that you set in step 2.

# **Step 4: Build Your Registry**

What will your registry look like, and what kinds of data will it help you track?

**1. Choose the right software for you** by considering the capability of your EMR/EHR or whether you will use external software, such as Excel, Access, or CDEMS.

**Resources:**

1. [Virginia HIT: Regional Extension Center](http://vhitrec.org/) – Offers HIT support for small practices, consulting services, access to discounted EHR software, and support for Meaningful Use attestation.
2. [Chronic Disease Registries Product Review](http://www.chcf.org/publications/2004/05/chronic-disease-registries-a-product-review) - California HealthCare Foundation (CHCF).
3. CHCF’s [Video comparing EHRs to Chronic Disease Management Systems](http://www.chcf.org/publications/2008/03/electronic-health-records-versus-chronic-disease-management-systems-a-quick-comparison).
4. Consider free electronic registry systems: [Chronic Disease Electronic Management System](http://www.cdems.com).

**2. Populate the registry** with patients, identified in step 3.2, and their related health factors identified in step 3.1.

# Step 5: Plan for Use of Your Registry

What new work-related tasks will your registry require, and who will do them?

**1. Define registry management tasks** that must be done when the registry is in use. For example:

- Keeping registry up-to-date with relevant patient data
- Outreach to patients found to have gaps in recommended preventive care
- Receiving returned calls after outreach has happened

**2. Assess workflow.** For example:

- - Who will be responsible for each registry task?
  - How often will the registry be updated?

**3. Develop a transition plan** by thinking through any necessary workflow changes, including the need to make time available for registry administration.

# Step 6: Implement Workflow Changes

How will the new tasks that your registry requires be implemented?

**1. Create tools** to help your staff complete registry-related tasks. These may include:

- - Scripts for calling patients who have missed appointments
  - Flow sheets, reminders in your EHR, or standing orders for visits

1. **Train staff** who will work with the registry by explaining its purpose, potential changes to their workflow, and review of registry-related tools.

**3. Provide information** for staff who will not work directly with the registry so they know what it is and can answer questions for others.

# **Step 7: Go Live**

Plan to pay extra attention to registry-related workflow the first week of implementation.

**Congratulations!** You have created a new tool for your practice that will enable you to work proactively to manage patient care.

**1. Have champions available** and ready to answer questions when you launch the registry.

**2. Review use of the registry** and related workflow at the end of the week to determine what changes, if any, you will have to make to your plan.

1. **Get patients involved.** Let patients in your practice know what you are doing to improve care delivery and help them understand goals that you have set for your practice.

# Step 8: Maintain Your Registry

**1. Plan for regular registry assessment.** A few weeks after first launcing your registry or “going live”, assess how use of the registry is going. Pay attention to the workflow of staff involved in working with the registry and how the registry affects other staff and patient care overall.

**2. Set a regular schedule for registry maintenance.** Your schedule might include steps such as updating the list of patients included in the registry, reviewing your targets and assessing any need for change, or setting new goals for the practice after you achieve your initial goals.

# Additional Resources

**Logistics**: Information on how to set up a registry and what a registry can do for your practice

- [Population Health Management – Diabetes Registry Guide](http://ndep.nih.gov/publications/PublicationDetail.aspx?PubId=114), National Diabetes Education Program
- [Registries: Powerful Tools to Track and Manage Chronic Disease](http://medicaleconomics.modernmedicine.com/medical-economics/news/registries-powerful-tools-track-manage-chronic-disease?page=full), Medical Economics, May 2013
- [Steps to Implementing a Patient Registry,](http://www.aafp.org/practice-management/pcmh/quality-care/patient-reg.html) AAFP
- [Peer Perspectives: What Registries Really Do](http://www.aafp.org/practice-management/pcmh/quality-care/patient-reg.html), Colorado Association of Family Physicians Newsletter
- [Registries Made Simple](http://www.aafp.org/fpm/2011/0500/p11.html%23fpm20110500p11-bt1), Family Practice Management, May 2011
- [Where is the patient in diabetes performance measures? The case for including patient-centered and self-management measures,](http://www.ncbi.nlm.nih.gov/pubmed/18445728) Diabetes Care, Glasgow et al., 2008
- [Care Process Model: Outpatient Management of Adult Diabetes Mellitus](https://kr.ihc.com/ext/Dcmnt?ncid=51061827), [Intermountain Healthcare](https://kr.ihc.com/ext/Dcmnt?ncid=51061827), April 2013

**Research** articles that provide information on potential benefits of implementing a registry

- Halladay, J. R., DeWalt, D. A., Wise, A., Qaqish, B., Reiter, K., Lee, S. Y., ... Donahue, K. E. (January 1, 2014). [More extensive implementation of the chronic care model is associated with better lipid control in diabetes.](http://www.jabfm.org/content/27/1/34.full) Journal of the American Board of Family Medicine, 27(1).
- Molina-Ortiz, E. I., Vega, A. C., & Calman, N. S. (July 1, 2012). [Patient Registries in Primary Care: Essential Element for Quality Improvement.](http://www.ncbi.nlm.nih.gov/pubmed/22786736) Mount Sinai Journal of Medicine: a Journal of Translational and Personalized Medicine, 79(4), 475–480.

Additional **incentive or recognition programs** related to developing a registry:

- [Diabetes Recognition Program](http://www.ncqa.org/Programs/Recognition/DiabetesRecognitionProgramDRP.aspx), NCQA
- [PQRS reporting](http://www.cms.gov/Medicare/Quality-Initiatives-Patient-Assessment-Instruments/PQRS/Registry-Reporting.html) through the [ABFM diabetes registry](https://www.theabfm.org/moc/pqrs.aspx), certified by CMS
